# Genetic characteristics and integration specificity of *Salmonella enterica* temperate phages

**DOI:** 10.3389/fmicb.2023.1199843

**Published:** 2023-08-01

**Authors:** Siqi Sun, Xianglilan Zhang

**Affiliations:** ^1^State Key Laboratory of Pathogen and Biosecurity, Beijing Institute of Microbiology and Epidemiology, Beijing, China; ^2^Department of Life Sciences and Technology, Beijing University of Chemical Technology, Chaoyang, Beijing, China

**Keywords:** temperate phage, *Salmonella enterica*, integrase, attachment site, correlation analysis, phylogenetic analysis, taxonomic classification

## Abstract

**Introduction:**

Temperate phages can engage in the horizontal transfer of functional genes to their bacterial hosts. Thus, their genetic material becomes an intimate part of bacterial genomes and plays essential roles in bacterial mutation and evolution. Specifically, temperate phages can naturally transmit genes by integrating their genomes into the bacterial host genomes via integrases. Our previous study showed that *Salmonella enterica* contains the largest number of temperate phages among all publicly available bacterial species. *S. enterica* is an important pathogen that can cause serious systemic infections and even fatalities.

**Methods:**

Initially, we extracted all *S. enterica* temperate phages from the extensively developed temperate phage database established in our previous study. Subsequently, we conducted an in-depth analysis of the genetic characteristics and integration specificity exhibited by these *S. enterica* temperate phages.

**Results:**

Here we identified 8,777 *S. enterica* temperate phages, all of which have integrases in their genomes. We found 491 non-redundant *S. enterica* temperate phage integrases (integrase entries). *S. enterica* temperate phage integrases were classified into three types: intA, intS, and phiRv2. Correlation analysis showed that the sequence lengths of *S. enterica* integrase and core regions of *attB* and *attP* were strongly correlated. Further phylogenetic analysis and taxonomic classification indicated that both the *S. enterica* temperate phage genomes and the integrase gene sequences were of high diversities.

**Discussion:**

Our work provides insight into the essential integration specificity and genetic diversity of *S. enterica* temperate phages. This study paves the way for a better understanding of the interactions between phages and *S. enterica*. By analyzing a large number of *S. enterica* temperate phages and their integrases, we provide valuable insights into the genetic diversity and prevalence of these elements. This knowledge has important implications for developing targeted therapeutic interventions, such as phage therapy, to combat *S. enterica* infections. By harnessing the lytic capabilities of temperate phages, they can be engineered or utilized in phage cocktails to specifically target and eradicate *S. enterica* strains, offering an alternative or complementary approach to traditional antibiotic treatments. Our study has implications for public health and holds potential significance in combating clinical infections caused by *S. enterica*.

## 1. Introduction

Temperate phages are important mobile genetic elements with significant impacts on essential bacterial cellular processes (Golding et al., 2021). During the lysogenic cycle of the temperate phage, the phage can integrate its own genes into the bacterial host genome via integrase, thus becoming an intrinsic part of the bacterium (Chen et al., 2020). Such an integration process can naturally transmit the important and essential genes of the temperate phage to its host, potentially increasing bacterial virulence and promoting antibiotic resistance (Harrison and Brockhurst, 2017; Monteiro et al., 2019).

Both integrases and their attachment sites in the respective phage and bacterial genomes are necessary components for temperate phage integration. During the integration process, the integrases encoded by the temperate phages first identify the core sequence regions of the phage attachment sites (*attP*) and the bacterial attachment sites (*attB*) and then mediate unidirectional site-specific recombination between them (Groth and Calos, 2004). Characterization of phage integrases and their attachment site (*att*) core regions is a crucial step to understand the phage integration process.

In a previous study, we observed that the *Salmonella enterica* genome contains the largest number of temperate phages compared to other sequenced bacterial species (Zhang et al., 2022). *Salmonella enterica* is a zoonotic pathogen of substantial concern to global human and animal health (Knodler and Elfenbein, 2019). It is a leading cause of morbidity and mortality in human beings worldwide. Identification of the genetic characteristics of integration specificity of *S. enterica* temperate phages will provide a foundation to both better understand the mechanisms of phage-bacteria interactions and to potentially modify phages to address the public health issues caused by *S. enterica* infections.

*Salmonella enterica* has been traditionally classified to different serotypes (serovars) and was reported that >2,500 serotypes cause most human infections (Jackson et al., 2013). Many researches focus on identifying and characterizing the temperate phages for specific *S. enterica* serotypes, such as phage mTmV in *S. enterica* serotype *Typhimurium* (*S. typhimurium*) ST34 (Tassinari et al., 2020), phage BIS20 in *S. typhimurium* (Sattar et al., 2022), phage ZCSE9 in *S. typhimurium* (Abdelsattar et al., 2023), phage SGP-C in *S. enterica serotype gallinarum biovar gallinarum* (*S. gallinarum*; Ilyas et al., 2022), phages vB_SenM-1, vB_SenM-2, vB_SenS-3 in 20 *S. enterica* serotypes, including *S. heidelberg*, *S. panama*, *S. typhimurium*, *S. cholerasuis*, *S. enteritidis*, *S. virchow*, etc. (Kosznik-Kwasnicka et al., 2020). However, to the best of our knowledge, no comprehensive research has been conducted on the large number of *S. enterica* temperate phages.

With the goal of systematically analyzing *S. enterica* temperate phages and gaining insight into their potential roles to *S. enterica* pathogenesis and evolution, we conducted an extensive examination of our previously published temperate phage genome dataset. From this dataset, we extracted 8,777 *S. enterica* temperate phages, each containing an integrase gene within their genomes. We further explored the genome characteristics, integration specificity, and genetic diversity of the temperate phages and their integrases.

Our analyses indicated that *S. enterica* temperate phages were primarily sourced from the U.S. and U.K. though also widely distributed across continental Europe. In total, the *S. enterica* temperate phages contained 491 different integrases within their genomes, which could be further categorized into three integrase types: intA, intS, and phiRv2. Strong correlations existed between the sequence lengths of integrases and the core regions of *attB* and *attP*. Phylogenetic analysis indicated that both the *S. enterica* temperate phage genomes and the integrase gene sequences were highly diverse. Most temperate phages showed high sequence similarities with phages within the bacterial genomes in the genera *Enterobacteriaceae*, *Pseudomonadaceae*, and *Vibrionaceae*.

Our work shed lights on the mosaicity and diversification of *S. enterica* temperate phages and their integrases at the genomic level. The identification of the sequence factors important for integration of *S. enterica* temperate phages revealed by our study will facilitate further research on the interactions between temperate phages and their hosts and the roles that temperate phages play in the evolution of bacteria. However, it is important to acknowledge some limitations of our work. First, while we extracted a large number of phages and integrases, the functional characterization of these elements was beyond the scope of this study. Further investigations are needed to understand the specific mechanisms and implications of these integrases in *S. enterica* pathogenesis and evolution. Second, our study focused primarily on the genomic level, and future studies should consider integrating other omics approaches to gain a more comprehensive understanding of the complex dynamics between temperate phages and their hosts.

## 2. Materials and methods

### 2.1. Identification of integrase sequences

This study identified a total of 8,777 temperate phages specifically associated with *S. enterica* from the comprehensive public and compromised temperate phage database generated in our previous study (Zhang et al., 2022). These temperate phages were detected from the bacterial next-generation sequencing data. In the previous study, we first used FastQC (Babraham Bioinformatics, 2010) and Trimmomatic (Bolger et al., 2014) for quality control and NGS reads filtering, followed by the steps of prophage detection and temperate phage detection (Zhang et al., 2022).

In this study, we identified the integrase sequences within the *S. enterica* temperate phage genome sequences by searching a customized integrase amino acid sequence database. To build such a customized database, we downloaded the amino acid sequences of all integrases listed on NCBI.^1^ The database contains 14,045 amino acid sequences of integrase, and its size is 6.3 megabytes (MB). We then added the customized integrase amino acid sequence database to Prokka v1.13 (Seemann, 2014) as a built-in database. We then annotated all *S. enterica* temperate phages using Prokka and extracted the annotated integrase sequences.

### 2.2. Extraction of the host information

We downloaded the host information of the *S. enterica* temperate phages from NCBI (See footnote 1) using their SRA numbers. Then, we wrote an in-house python script to extract the host information, including sample collection time, host source, and geographic distribution.

### 2.3. Generation of temperate phage and integrase entries

We used CD-HIT v4.81 (Fu et al., 2012) to group the sequence of each temperate phage and its reverse complement into a single temperate phage entry. Each temperate phage entry contains the temperate phage genome sequences with 100% identity and 100% coverage. The same was done with the sequence of each integrase and its reverse complement. Strict CD-HIT parameters were used. In particular, the parameter c was set to 1, representing 100% similarity between any two sequences; the parameters AL and AS were both set to 0, indicating that the two sequences matched exactly without any non-aligned parts.

### 2.4. Phylogenetic analysis of temperate phage and integrase entries

We first used MAFFT v7.505 (Katoh, 2002) to generate multiple sequence alignments of temperate phage and integrase entries, respectively. In order to focus on obtaining sequence clusters rather than evolution rates, we employed the neighbor-joining (NJ) method for constructing the phylogenetic tree due to its efficiency. Specifically, we used TreeBest (Vilella et al., 2009) software to build the neighbor-joining trees based on the temperate phage nucleotide sequence alignments and the integrase amino acid sequence alignments. Default parameters were used, except for setting the bootstrap value to 1,000. The phylogenetic trees were displayed using the online tool ChiPlot.^3^

### 2.5. Taxonomic classification of *Salmonella enterica* temperate phages

The putative open reading frames (ORFs) of all 3,857 *S. enterica* temperate phages were identified and translated using Prodigal v1.13 (Hyatt et al., 2010). Then, the putative temperate phage ORFs were input into vContact2 to perform taxonomic clustering using the ProkaryoticViralRefSeq94-Merged database with default parameters (Bin Jang et al., 2019).

### 2.6. Correlation analysis of *Salmonella enterica* temperate phages, integrases, and core regions of attachment sites *attB* and *attP*

We used Spearman correlation coefficients (rs) to assess the relationships of both sequence lengths and GC content among temperate phages, integrases, and core attachment site regions. We assessed the correlation based on both rs and *p*-values. If rs > 0.3 and *p* < 0.05, we considered the two variables to be correlated. The rs was calculated using the R package ggpubr, and the figures were generated using the R package ggplot2.

### 2.7. Analysis of *Salmonella enterica* serotypes infected by the *Salmonella enterica* temperate phages

To be precise, we consider temperate phages that have been reported to integrate into their host genomes. This indicates that a specific *S. enterica* serotype has been found to be infected by these temperate phages. Integration refers to the inclusion of temperate phage genome sequences within the host genomes, illustrating that they share sequence similarities. Particularly, we used BLASTn to align all the 3,857 *S. enterica* temperate phage entries (non-redundant phage genome sequences) against nucleotide database of NCBI. We then extracted all the temperate phage genome sequences aligned to the *S. enterica* serotypes genome sequences (with sequence identify > 80% and sequence coverage > 80%).

## 3. Results

### 3.1. Genomic characteristics of *Salmonella enterica* temperate phages and their integrases

**Figure 1:**
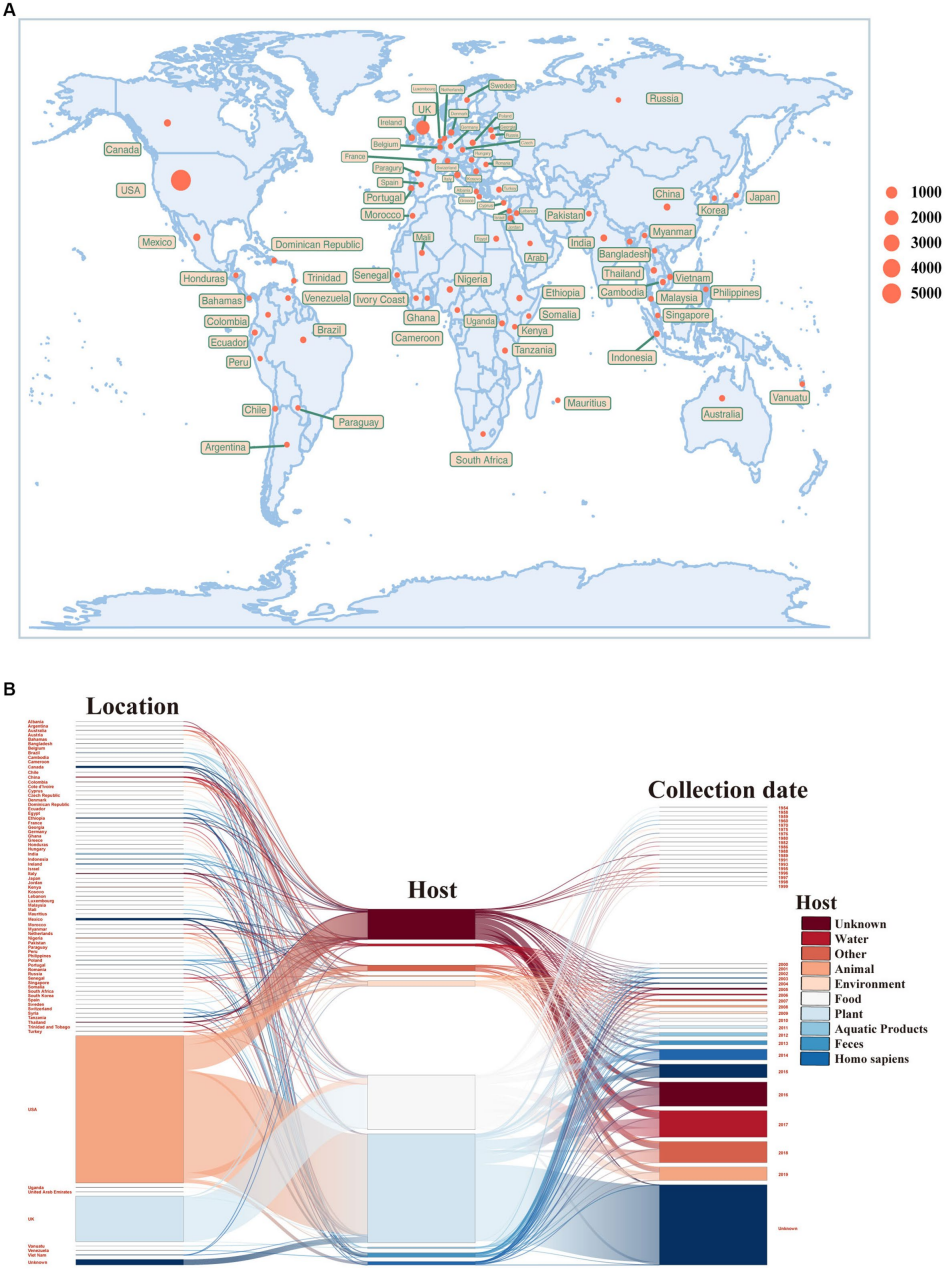
Sample information of *Salmonella enterica* temperate phages. **(A)** Geographical distribution of *S. enterica* temperate phages. The size of the sampling point indicates the number of samples. **(B)** Sankey diagram of sample sources of *S. enterica* temperate phages. The bars from left to right indicate geographic location, host source, and sampling time, respectively.

To begin our study, we obtained an extensive collection of *S. enterica* temperate phages from a publicly accessible and comprehensive temperate phage database that we had previously developed (Zhang et al., 2022). Within this database, we meticulously identified attachment site core sequences at both ends, facilitating the precise delimitation of prophage sequences. Leveraging the wealth of data provided by this database, we proceeded to conduct an exhaustive analysis encompassing a total of 8,777 *S. enterica* temperate phages, including the examination of their integrases. The *S. enterica* hosts were widely distributed across six continents, though the majority of samples were acquired from the U.S. and the U.K. (Figure 1A; Supplementary Table 2). The *S. enterica* studied here were primarily sampled from human hosts (Figure 1B; Supplementary Table 2).

**Figure 2:**
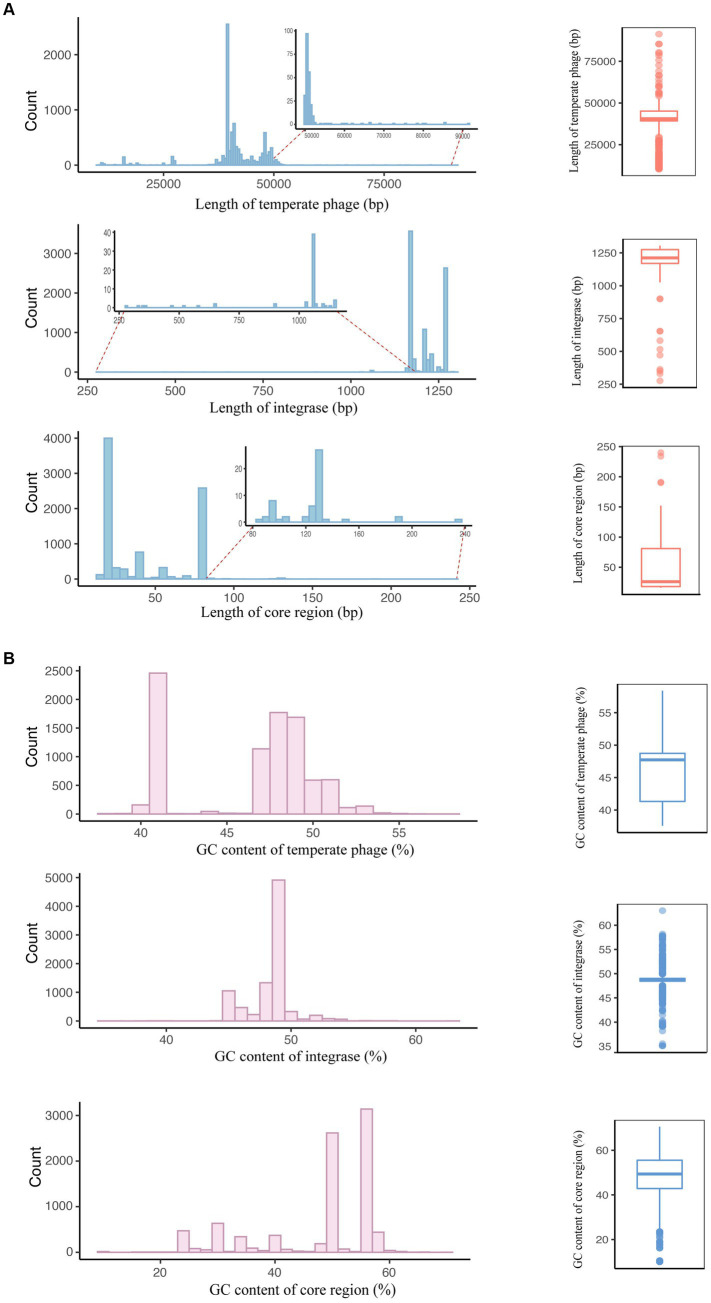
Basic sequence characteristics of temperate phages, integrases, and attachment site core regions. **(A)** Sequence length distributions of temperate phages, integrases, and the core regions of *attB* and *attP*. The left panel depicts the number of sequences with the same length. The right panel illustrates the distributions of the sequence lengths. **(B)** GC content distributions of temperate phages, integrases, and the core regions of *attB* and *attP*. The left panel depicts the number of sequences with the same GC content. The right panel illustrates the distributions of GC content.

Considering that sequence length and GC content are fundamental characteristics of genomes, our initial analysis focused on examining the distribution of lengths and GC contents among temperate phages of *S. enterica*. To be specific, the sequence lengths of the *S. enterica* temperate phages and integrases covered a relatively narrow range (Figure 2A; Supplementary Table 1), with most temperate phages between 35,000 and 40,000 bp in length and most integrases between 1,170 and 1,275 bp. Similarly, the GC content of these elements covered a narrow range, from 42% to 48% GC content in temperate phages and 48% to 49% in the integrases (Figure 2B; Supplementary Table 1).

Even though the number of extremely large (>70,000 bp) temperate phages is very small, their presence is noteworthy. These phages make up only 0.09% (8/8,777) of the temperate phage genome sequences identified in our previous study, where these temperate phages were extracted from bacterial high-throughput sequencing data based on their spontaneous induction (Zhang et al., 2022). When temperate phages are induced to a particular concentration from their host strains and their circular phage genome sequences are detected, we can confidently recognize these sequences as complete temperate phage genome sequences.

In other studies, the reported genome sizes for phages induced from STEC (Shiga toxin-producing *Escherichia coli*) belonging to serogroups O26, O29, and O157 ranged from 47 to 70 kb (Willshaw et al., 1987; Rietra et al., 1989; Schmidt, 2001). Additionally, prophage genomes in Baltic Sea bacterioplankton isolates were found to vary in size from 8 to 87 kb, with 57% falling within the 35–45 kb range (Leitet et al., 2006). These findings underscore the diversity and range of temperate phage genome sizes, with some temperate phages exhibiting notably larger genomes.

It has been observed that temperate phages found in various hosts may possess identical genome sequences. Consequently, we consider these temperate phage genome sequences, exhibiting 100% identity and 100% coverage, as a single temperate phage entry (non-redundant phage genome sequence). In total, we identified unique 3,857 temperate phage entries (non-redundant phage genome sequences) and 491 unique integrase entries (non-redundant integrase gene sequences).

### 3.2. The correlation among *Salmonella enterica* temperate phages, integrases, and the core regions of the attachment sites *attB* and *attP*

**Figure 3:**
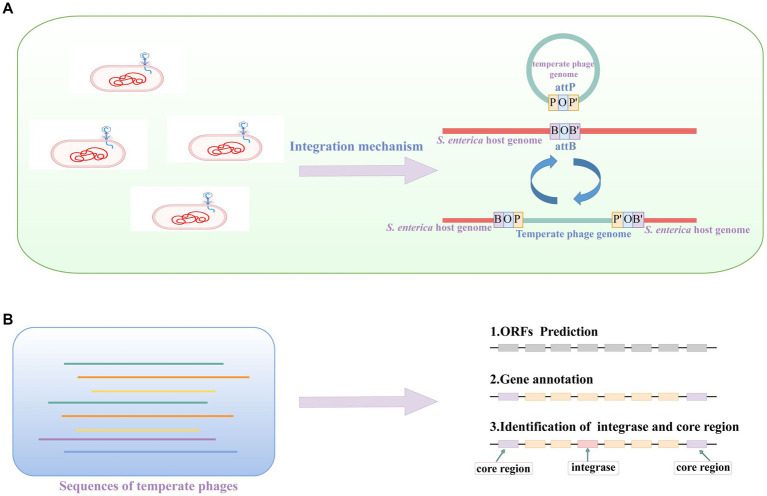
The illustration of temperate phage induction, core region and integrase. **(A)** Temperate phage induction and integration processes. The letter B represent the sequence on host bacteria, the letter P shows the sequence of temperate phage, and the letter O means the core regions of the attB and attP sites. **(B)** The identification of the core region and integrase of a temperate phage. These temperate phage genome sequences are from the database we generated in a previous study (Zhang et al., 2022).

As depicted in the upper panel of Figure 3A, the core regions of the attachment sites *attB* and *attP* consist of identical sequences shared by both sites. A temperate phage can use integrase to integrate into host genome. In our previous study, we provided the complete temperate phage genomes (Zhang et al., 2022). From these genomes, we further extracted the core region and integrase for each phage (Figure 3B). To accomplish this, we initially predicted the ORFs and then proceeded with gene annotation. Finally, we successfully identified the core region and integrase of each temperate phage genome.

We used Spearman correlation coefficients (rs) to measure correlations among the sequence lengths of all three aforementioned elements, namely temperate phages, integrases, and attachment site core regions. We evaluated the correlation between variables using both rs (correlation coefficient) and value of ps. To determine a correlation, we considered variables to be correlated if the rs value exceeded 0.3 and the corresponding *p*-value was less than 0.05.

**Figure 4:**
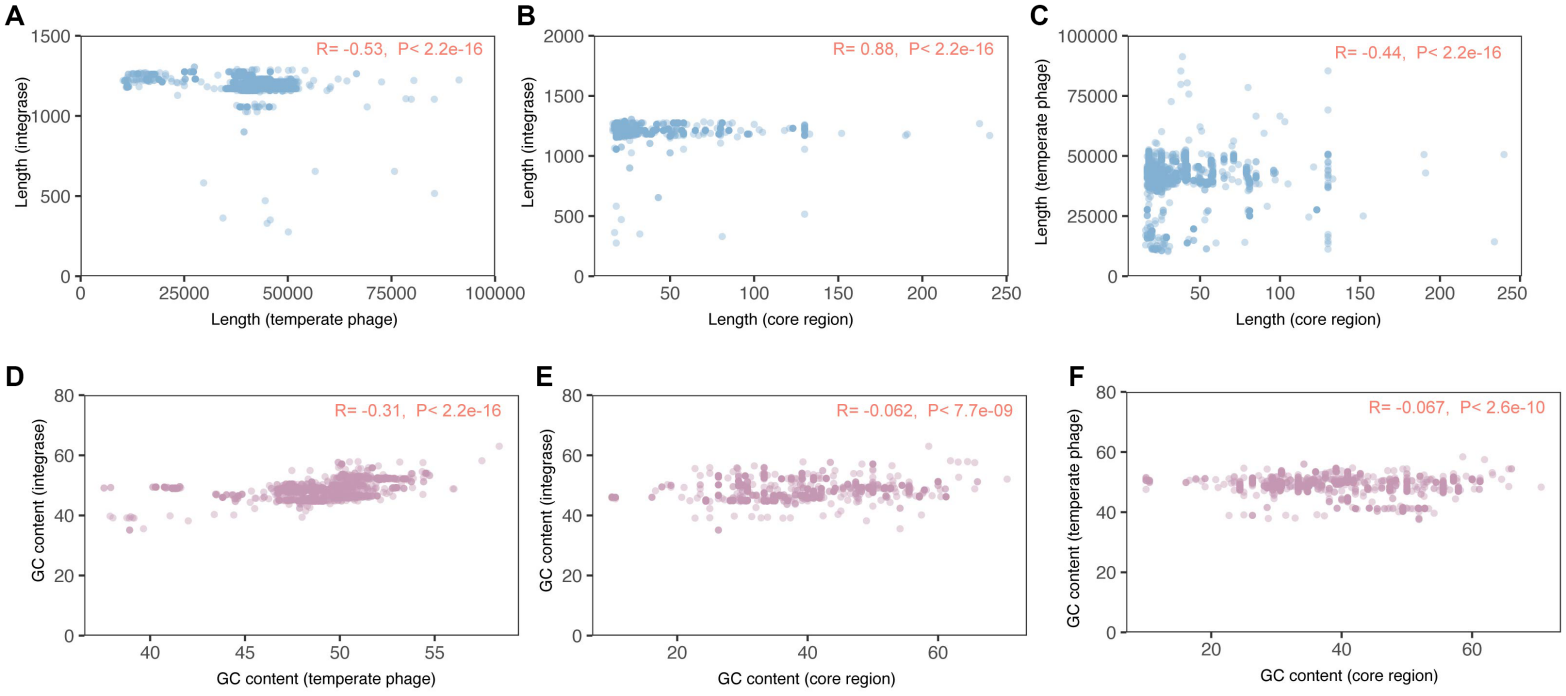
Correlations among the sequences of temperate phage, integrase, and attachment site core region sequences. R > 0.3 and *p-*value < 0.05 indicate the two elements were correlated. **(A–C)** Correlations among sequence lengths of temperate phages, integrases, and *attB* and *attP* core regions. **(D–F)** Correlations among GC content of temperate phages, integrases, and *attB* and *attP* core regions.

In particular, we observed a strong positive association between the sequence lengths of integrases and the core regions of *attB* and *attP* (R = 0.88, *p* < 2.2e − 16, Figure 4B; Supplementary Table 1). This positive association suggests that longer integrase sequences are correlated with longer core regions of attachment sites. Conversely, the correlations between the sequence lengths of temperate phages and the other two elements were both modest and negative. Specifically, there was a negative correlation between temperate phages and integrases (R = −0.53, *p* < 2.2e − 16, Figure 4A; Supplementary Table 1), as well as between temperate phages and attachment site core regions (R = −0.44, *p* < 2.2e − 16, Figure 4C; Supplementary Table 1). This negative correlation indicates that longer temperate phage sequences are associated with shorter integrase sequences/core regions of attachment sites.

Additionally, we found a modest and negative correlation between the GC content of integrases and temperate phages (R = −0.31, *p* < 2.2e − 16, Figure 4D; Supplementary Table 1). This negative correlation suggests that as the GC content of integrases increases, the GC content of temperate phages tends to decrease, and vice versa. However, we did not observe monotonic associations between the GC content of core regions and either attachment site core regions (R = −0.062, *p* < 7.7e − 09, Figure 4E; Supplementary Table 1) or integrases (R = −0.067, *p* < 2.6e − 10, Figure 4F; Supplementary Table 1). These non-monotonic associations indicate that changes in the GC content of a temperate phage or its integrase would not significantly impact the GC content of the core regions of *attB* and *attP*.

**Figure 5:**
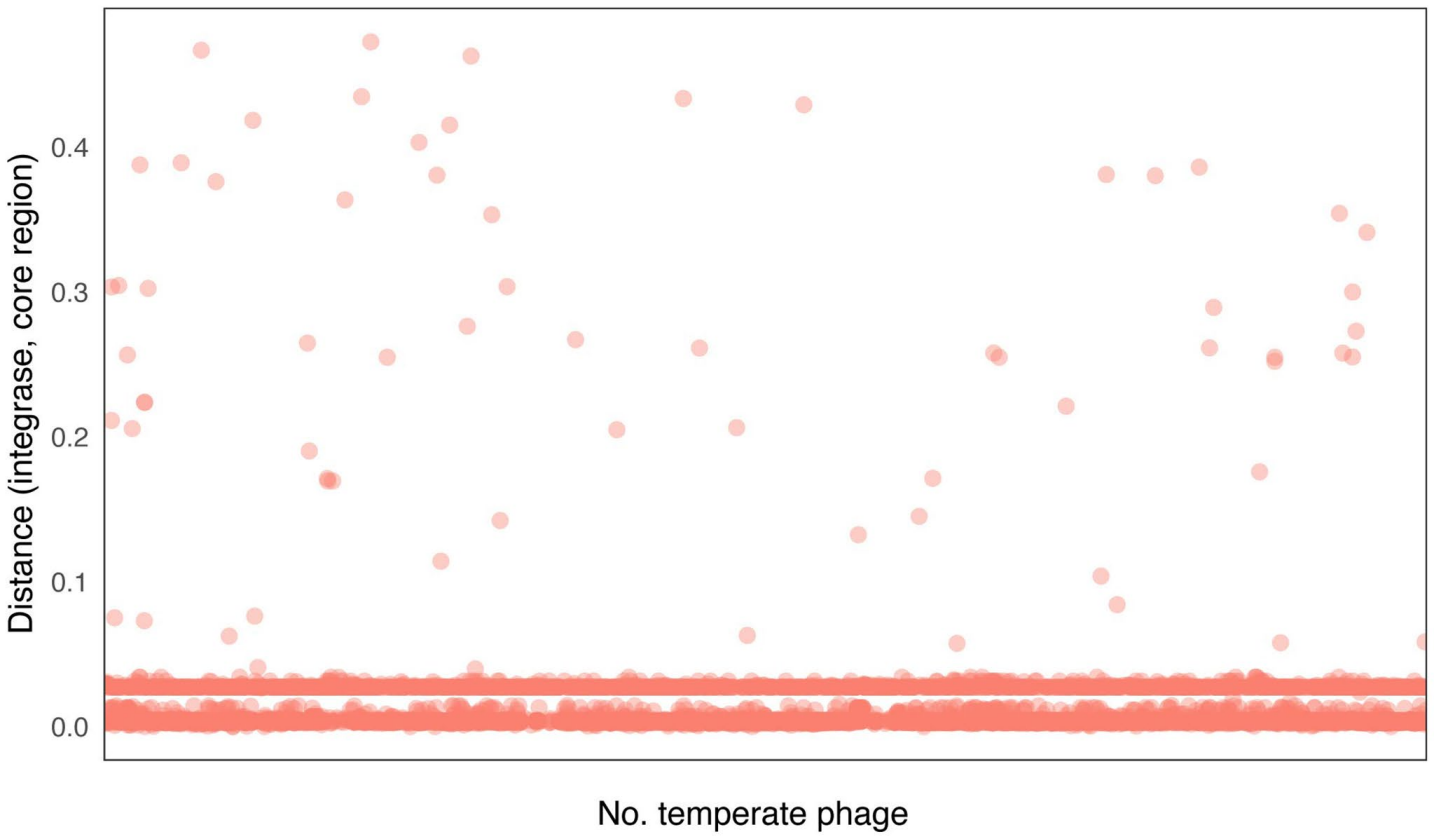
Distribution of distances between integrase sequences and attachment site core region sequences. Distance (integrase, core region) = |Position (integrase) – Position (core region)|/Length (temperate phage).

Our findings indicate that integrases typically localize in close proximity to attachment site core regions (Figure 5; Supplementary Table 1), which aligns with previous studies (Karaolis et al., 1998; Novick and Subedi, 2007; Tanaka et al., 2009). To be more precise, the relative distance between the integrase and the core regions of the attachment site is commonly found within two distinct ranges. Approximately 58% of temperate phages have lengths ranging from 102 to 215 bp, whereas 34% of temperate phages have lengths ranging from 774 to 1,068 bp. The relative distances are concentrated in the ranges of 0.002–0.005 and 0.027–0.028, respectively. This concentration of relative distances suggests that there are specific preferred positioning patterns for integrases in relation to the attachment site core regions. Our findings indicate that integrases tend to localize near the core regions of attachment sites. The concentration of relative distances within specific ranges suggests preferred positioning patterns, further highlighting the functional relationship between integrases and the attachment site core regions.

### 3.3. The sequence varieties and genetic characteristics of *Salmonella enterica* temperate phages and their integrases

Each temperate phage identified in this study contained a single integrase. That is, 8,777 *S. enterica* temperate phages contained 8,777 integrases. As mentioned in the previous section (3.1), we classify these temperate phage genome sequences, which display 100% identity and 100% coverage, as a phage genome sequence entry (non-redundant temperate phage sequences). In total, these 8,777 *S. enterica* temperate phages were grouped into 3,857 unique *S. enterica* temperate phage entries (non-redundant temperate phages sequences). The 8,777 *S. enterica* integrases were grouped into 491 unique *S. enterica* integrase entries (non-redundant integrase sequences).

By aligning the 491 unique *S. enterica* integrases to the custom integrase database (Methods and Materials 2.1), all 491 integrases were classified into three types: intA, intS, and phiRv2 (Supplementary Table 4). The intS is a phage P4-type symbiosis island integrase protein, which is required for integration and excision of the island (Ramsay et al., 2006). The intA is an Stx-like integrase protein (Hochhut et al., 2006). Shiga toxin (Stx) and Stx-like toxins are normally found in various lambdoid phages that can integrate into multiple sites within the Shigella species and select *Escherichia coli* pathovars (O’Brien et al., 1984, 1989). The phiRv2 has previously been detected in *Mycobacterium tuberculosis* H37Rv genome (Mahadevan et al., 1998), encoding a tyrosine recombinase (Bibb and Hatfull, 2002).

Most of the integrases (63.7%) were classified as intS, while 35.8% of the integrases were classified as intA and only two were classified as phiRv2. It is noted that intA is normally found in Shiga and *E. coli* species, and phiRv2 is normally found in *M. tuberculosis*. The finding of intA and phiRv2 integrases in *S. enterica* temperate phages implies a potential for horizontal gene transfer and genetic diversification among the different species of bacterial populations; while the low representation of phiRv2 integrases suggests a lesser involvement of the PhiRv2 prophage in these bacterial populations.

**Figure 6:**
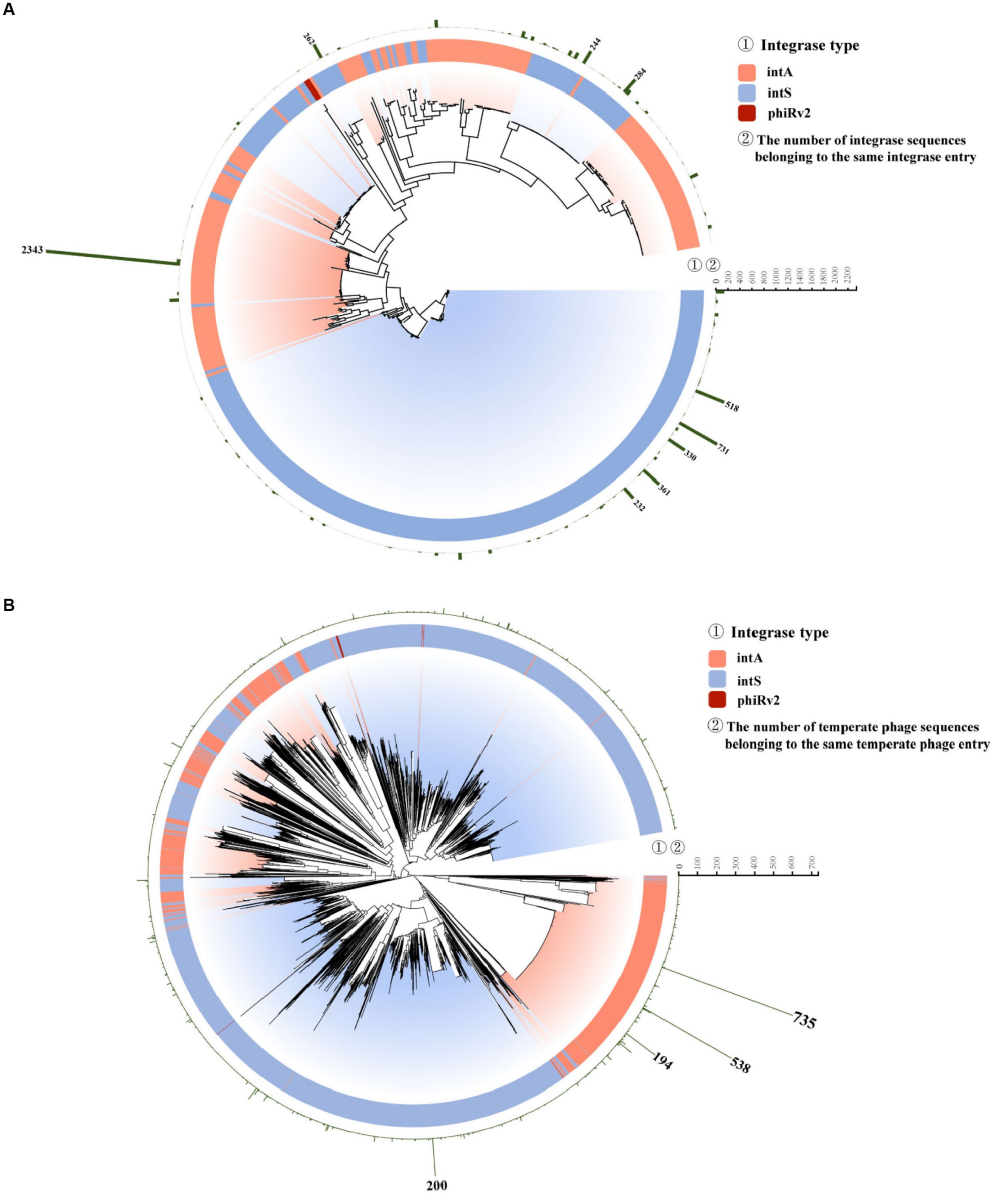
Phylogenetic diversity of unique integrase and temperate phage sequences. **(A)** Neighbor-joining (NJ) tree of 491 integrase entries. The inner string indicates the type of integrase. The outer string indicates the number of integrases in each entry. **(B)** Neighbor-joining (NJ) tree of all 3,857 temperate phage entries. The inner string indicates the type of integrase. The outer string indicates the number of temperate phages in each entry.

Phylogenetically, most integrases of type intS were grouped in several closest clades (Figure 6A, bottom; Supplementary Table 4). Integrase entry No. 10, of the intA type, had the largest number (*n* = 2,343) of integrase sequences (Figure 6A; Supplementary Table 4). The temperate phages within integrase entry No.10 were mostly identified from bacterial hosts isolated from humans, sampled between 1954 and 2019. These were sourced from 20 different countries, primarily the U.K. (*n* = 1,109) and the U.S. (*n* = 1,053; Supplementary Tables 4, 5).

In the phylogenetic tree of *S. enterica* temperate phages, we labeled each temperate phage sequence according to its integrase type (Figure 6B; Supplementary Table 3). The 2,343 integrases in integrase entry No. 10 were from 2,343 temperate phages being grouped into 325 unique temperate phage entries. Within these 325 unique temperate phage entries, phage entry No. 2869 contained the largest number of temperate phage sequences (*n* = 735, Figure 6B; Supplementary Tables 3, 5). The phage entry No. 2869 had the highest number of human hosts, and these were sampled between 2009 and 2019 and sourced from eight countries, primarily the U.K. (*n* = 653; Supplementary Tables 2, 3).

### 3.4. Taxonomic classification of *Salmonella enterica* temperate phages

All 8,777 *S. enterica* temperate phage genomes were taxonomically grouped with phages from publicly available databases. In particular, we used vContact2 (Bin Jang et al., 2019) to cluster the temperate phage genome sequences with the reference virus genomes, and then build gene-sharing networks between the temperate phages and reference viruses based on shared protein clusters (PCs). In this way, vContact2 provided the taxon assignments to the *S. enterica* temperate phage in our study. Specifically, we utilized vContact2 to cluster the *S. enterica* temperate phage genome sequences with the reference virus genomes. Then gene-sharing networks between the temperate phages and reference viruses were constructed based on shared protein clusters (PCs). Through this approach, vContact2 enabled the taxonomic assignment of all the *S. enterica* temperate phages in our study.

As a result, the 8,777 *S. enterica* temperate phage genomes had similarities with 269 phages in public databases. The 269 phages were grouped into 21 host families (Supplementary Table 7), with most grouped with *Enterobacteriaceae* (*n* = 122, 45%), *Pseudomonadaceae* (*n* = 62, 23%), and *Vibrionaceae* (*n* = 13, 4%). That is, most *S. enterica* temperate phages had sequence similarities with phages from *Enterobacteriaceae*, *Pseudomonadaceae*, and *Vibrionaceae*. On the other hand, the 269 phages include 146 different phages, such as phi-like, Bcc-specific (Bccp), Mu-like, P2, Sf phages (Supplementary Table 7). It illustrates that the studied population of the 8,777 *S. enterica* temperate phages is diverse and rich, with 146 distinct types identified from the total of 269 phages. This diversity suggests a wide range of potential interactions and impacts on the bacterial community within the studied environment.

**Figure 7:**
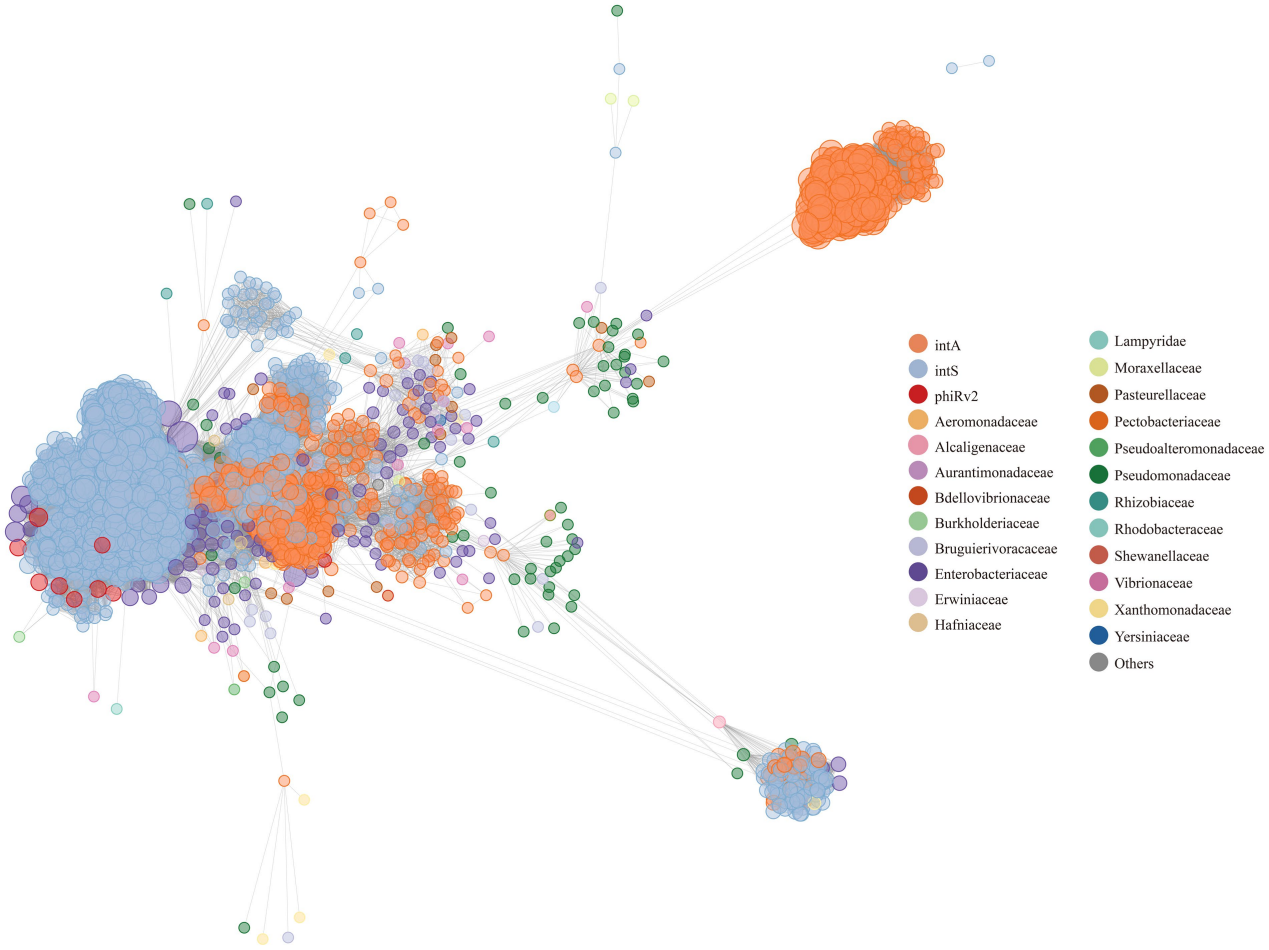
A gene-sharing network of 3,857 unique temperate phages. The genomes of the 3,857 unique phage entries were compared with the bacterial virus genomes retrieved from ViralRefSeq94-Merged database. The phage genomes in our study are colored by their integrase types, while the phage gene sequences from the database are colored according to their family level. Each node represents a phage gene sequence, while each edge displays the similarity between any two phage gene sequences. The larger the node is, the more similarity it has with other phages.

**Figure 8:**
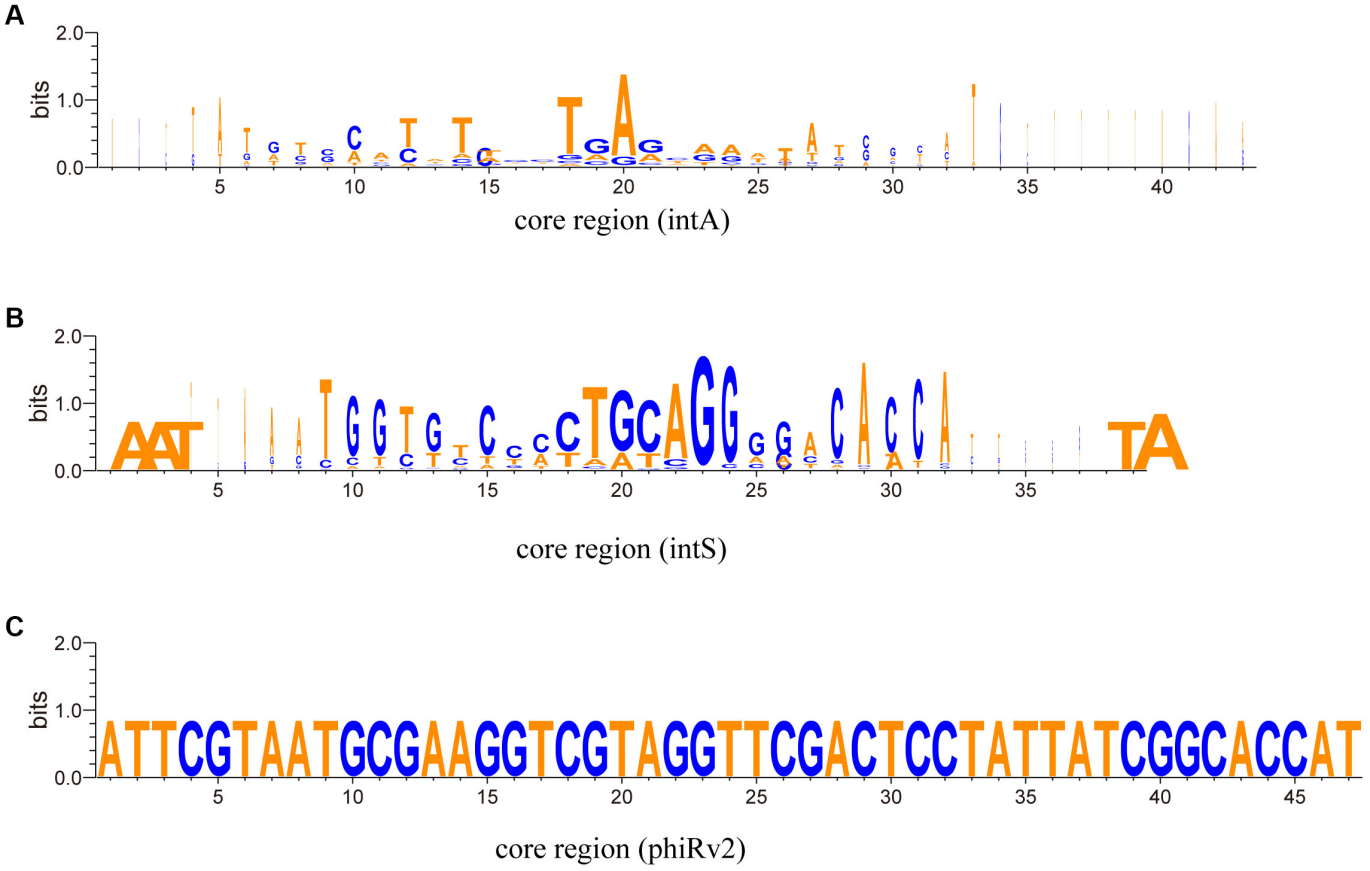
Aligned Weblogos depicting the core region sequences of various integrase types. The X-axis represents the nucleic acid base position, while the Y-axis quantifies the sequence conservation at each position (measured in bits). **(A)** The Weblogo analysis of the core region sequences of integrase intA. **(B)** The Weblogo analysis of the core region sequences of integrase intB. **(C)** The Weblogo analysis of the core region sequences of integrase phiRv2.

The taxonomic results show that from the aspect of integrase types, some temperate phages with intA and intS integrases were taxonomically clustered, respectively (Figure 7, the two clusters on the right; Supplementary Tables 6, 7); while some phages with different integrase types were taxonomically mixed together (Figure 7, left and middle; Supplementary Tables 6, 7). We conducted an analysis of the nucleic acid sequences of the core regions of *attP* and *attB* sites (Figure 8; Supplementary Table 1). The findings reveal that temperate phages with intA integrase exhibit consistent core regions at positions 18 to 21 (TGAG), while those with intS integrase show conservation at starting positions with AAT, ending positions with TA, and middle positions from 19 to 24 (TGCAGG). Furthermore, phages with phiRv2 integrase display consistency at all positions. These results highlight a correlation between the core region sequences and the integrase types, as evidenced by distinct conserved positions for different integrase types.

### 3.5. *Salmonella enterica* serotypes infected by the *Salmonella enterica* temperate phage genome sequences

Among the 3,857 non-redundant *S. enterica* temperate phage genome sequences, a total of 40 *S. enterica* serotypes are susceptible to be infected by temperate phages (Supplementary Table 8), suggesting a diverse range of phage-host interactions within the *S. enterica* species. The predominant serotype is *S. enterica* serotype (serovar) *Enteritidis* (*S. enteritidis*), being infected by 330 *S. enterica* temperate phages (Supplementary Table 8). *Salmonella enteritidis* is a globally recognized serotype with significant public health implications (Whistler et al., 2018). It is widely recognized as one of the most prevalent strains responsible for human infections worldwide second to *S. typhimurium* (Galanis et al., 2006). *Salmonella typhimurium* can be infected by 46 temperate phages in our study.

**Figure 9:**
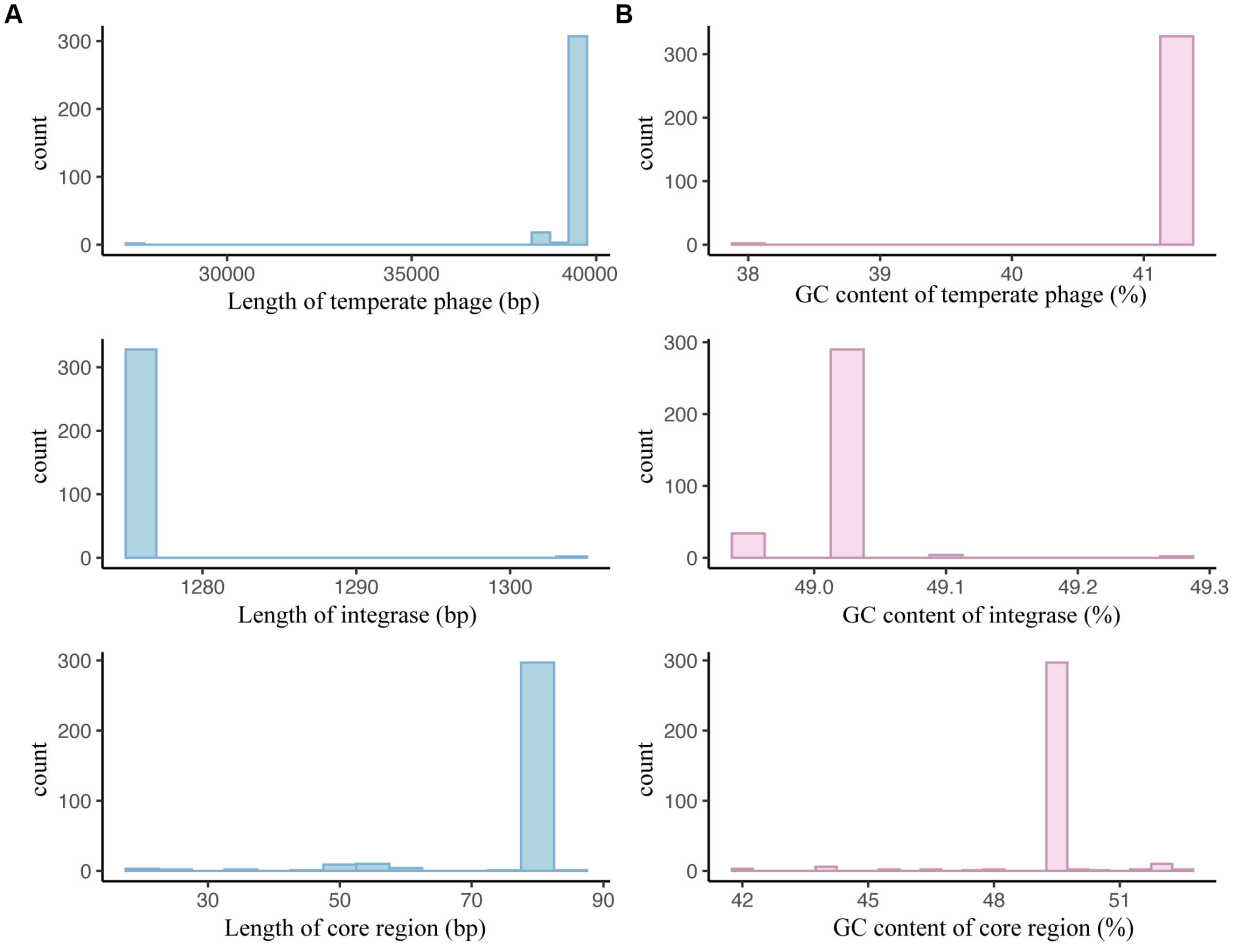
Basic sequence characteristics of *Salmonella enteritidis* temperate phages, integrases, and attachment site core regions. **(A)** Sequence length distributions of *S. enteritidis* temperate phages, integrases, and the core regions of *attB* and *attP*. **(B)** GC content distributions of *S. enteritidis* temperate phages, integrases, and the core regions of *attB* and *attP*.

Regarding the sequence length and GC content, we observed a relatively narrow range for *S. enteritidis* temperate phages, integrases, and core regions (Figure 9; Supplementary Table 9). Most temperate phages had lengths between 39,267 bp and 39,279 bp, integrases had a length of 1,275 bp, and core regions had a length of 81 bp (Figure 9A; Supplementary Table 9). Similarly, their GC content exhibited a narrow range, with most temperate phages ranging from 41.32% to 41.33% GC content, integrases at 49.02%, and core regions at 49.38% (Figure 9B; Supplementary Table 9).

**Figure 10:**
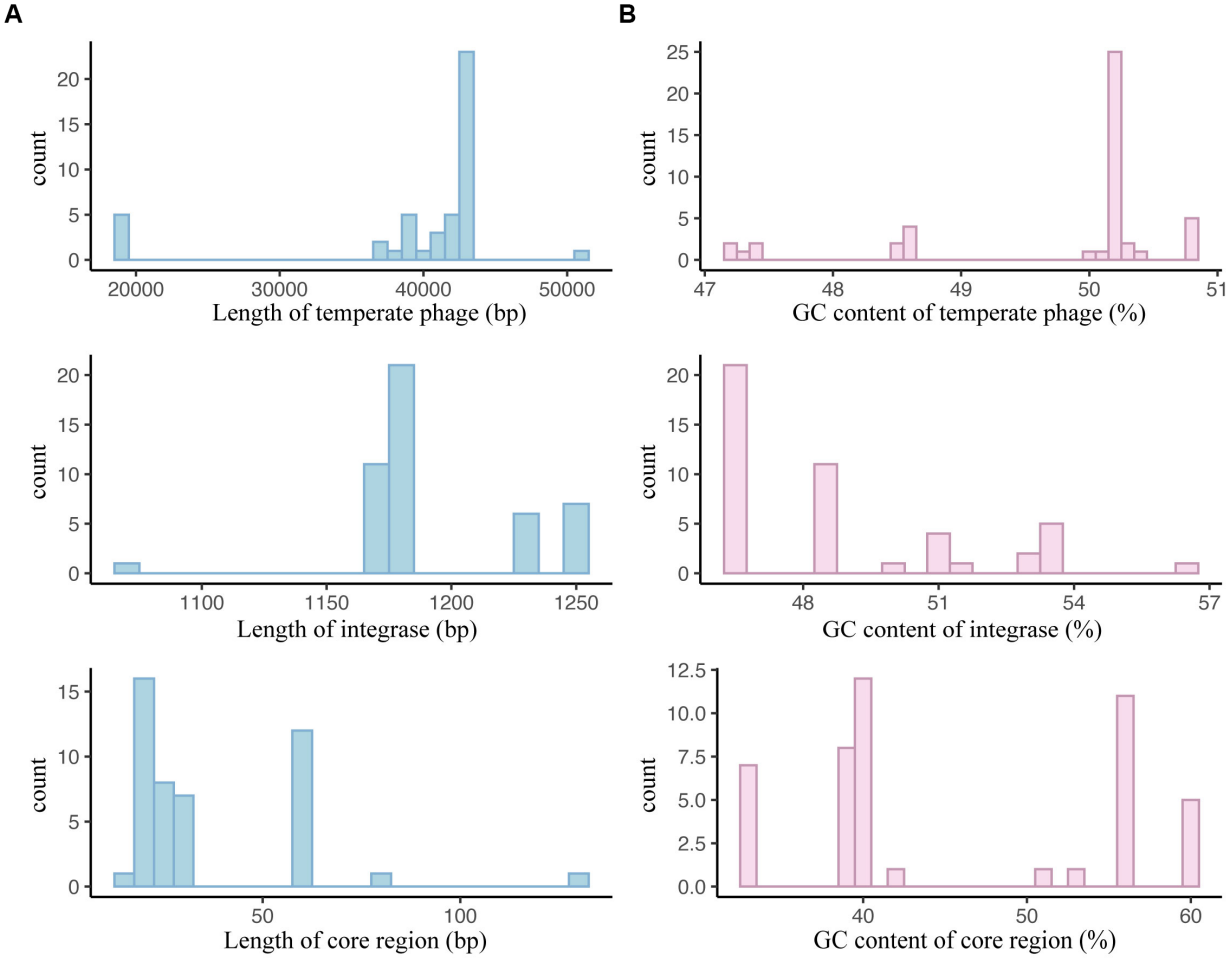
Basic sequence characteristics of *Salmonella typhimurium* temperate phages, integrases, and attachment site core regions. **(A)** Sequence length distributions of *S. typhimurium* temperate phages, integrases, and the core regions of *attB* and *attP*. **(B)** GC content distributions of *S. typhimurium* temperate phages, integrases, and the core regions of *attB* and *attP*.

For *S. typhimurium* temperate phages, their sequence lengths primarily fell within the 38,866 bp to 42,575 bp, integrases ranged from 1,173 bp to 1,227 bp, and core regions from 18 bp to 58 bp (Figure 10A; Supplementary Table 10). The GC contents of temperate phage genomes, integrases, and core regions were mainly between 50.05% to 50.23%, 46.70% to 51.18%, and 39.13% to 55.56%, respectively (Figure 10B; Supplementary Table 10).

## 4. Discussion

*Salmonella enterica* is an important pathogen that can cause life-threatening diseases in humans and animals. The temperate phages of *S. enterica* play essential roles in bacterial evolution and pathogenicity, especially in the horizontal transfer of virulence factors and antibiotic resistance genes. Our previous study also found *S. enterica* had the largest number of temperate phages of all sampled bacterial species (Davies et al., 2016).

This study focused on basic genetic characteristics and integration specificity of *S. enterica* temperate phages. We analyzed 8,777 *S. enterica* temperate phage genomes and identified the integrases contained within each one. The hosts of the *S. enterica* temperate phages were widely distributed all over the world (Figure 1; Supplementary Table 2). Probably due to sampling bias, these temperate phages were mainly distributed in the U.S. and the U.K., and primarily sourced from humans. The presence of unique phage types or variants specific to other regions might be missed due to the limited geographical representation. This could result in an incomplete understanding of the taxonomic composition and functional characteristics of *S. enterica* temperate phages on a global scale. Future studies should aim to expand the geographical representation, including diverse ecological settings, to obtain a more comprehensive assessment of the global distribution and diversity of *S. enterica* temperate phages. From the basic sequence characteristics of the temperate phages, we found that the sequence lengths of phages, integrases, and the core regions of *attB* and *attP* had modestly negative (phages vs. integrases, and phages vs. core regions) correlations (Figure 4; Supplementary Table 1). The negative correlations in sequence lengths suggest that there might be a coordinated evolution between phages, integrases, and the core regions of *attB* and *attP*. This coordination could indicate functional or structural dependencies among these elements during phage integration. It is possible that maintaining specific length ratios between these sequences is important for the efficient and stable integration of the phage genome into the host bacterial chromosome.

We also discovered a strong positive correlation between the lengths of integrases and the core regions (Figure 4; Supplementary Table 1). It is reported that the efficiency of integrase reduces at shorter *attB* length (Ghosh et al., 2003). Our study found that the phage with shorter integrase normally has shorter attachment site core region. Thus, we hypothesized that the reduced integrase efficiency may due to its shorter length.

The GC content between phages and integrases was weakly correlated (Figure 4; Supplementary Table 1). This suggests that there might not be a strong relationship between the GC content of phages and integrases, indicating that factors other than GC content might be driving their co-occurrence or co-evolution.

Also our results showed that the integrases of *S. enterica* temperate phages were often localized around attachment sites (Figure 5; Supplementary Table 1), which is consistent with the previous studies (Huang et al., 2019; Yarnall et al., 2022). The localization of integrases near attachment sites suggests a potential functional advantage in terms of integration efficiency. By being in close proximity to the attachment sites, integrases can readily access and interact with the specific sequences required for recombination. This proximity facilitates the efficient recognition and binding of the attachment site core regions by the integrase, promoting successful integration of the phage DNA into the host genome.

Our analysis identified 3,857 unique *S. enterica* temperate phage sequences with integrases from three categories: intA (27.15%, 1,047/3,857), intS (72.62%, 2,801/3,857), and phiRv2 (0.23%, 9/3,857).

The presence of intA integrases in *Shiga* and *E. coli* species, as well as phiRv2 integrases in *M. tuberculosis*, has been commonly observed. However, the identification of intA and phiRv2 integrases in temperate phages of *S. enterica* suggests the possibility of horizontal gene transfer and genetic diversification among different bacterial species. This horizontal gene transfer could contribute to the acquisition of new genetic elements, virulence factors, and the genetic diversification within bacterial populations, leading to the emergence of novel traits, pathogenicity, or phenotypes.

On the other hand, the relatively low representation of phiRv2 integrases suggests a lesser involvement of the PhiRv2 prophage in the bacterial populations of *S. enterica*. This implies that the transfer of genetic material associated with the PhiRv2 prophage is less frequent or less successful in *S. enterica* compared to its native host, *M. tuberculosis*.

The presence of intA and phiRv2 integrases in *S. enterica* temperate phages highlights the potential for horizontal gene transfer and genetic exchange among different bacterial species. The lower representation of phiRv2 integrases suggests a less prominent role of the PhiRv prophage in *S. enterica* populations compared to its association with *M. tuberculosis*.

Phylogenetically, most integrases of type intS tend to be grouped together (Figure 6A, bottom; Supplementary Table 4). The integrase sequences of type phiRv2 (Figure 6A, in red; Supplementary Table 4) had low similarity with the sequences of types intA and intS. While most phages have the integrase type intS (63.7%) and intA (35.8%), only two phages with type phiRv2 (0.5%).

On the other hand, the temperate phages with integrases intA and intS were mixed in both (Figure 6B; Supplementary Table 3). Taxonomically, some *S. enterica* temperate phages with the same integrase type tended to fall within the same taxonomic groups (Figure 7, two clusters on the right, Supplementary Tables 6, 7), while some phages with different integrase types were mixed together (Figure 7, left and middle; Supplementary Tables 6, 7). The results suggested that besides the integrase type, other factors may also influence the evolution of *S. enterica* temperate phages, which need to be further explored. In terms of sequence similarity, the *S. enterica* temperate phage sequences were highly similar to the phage sequences of *Enterobacteriaceae*, *Pseudomonadaceae*, and *Vibrionaceae*.

Among the *S. enterica* serotypes susceptible to *S. enterica* temperate phages, *S. enteritidis* stands out as the dominant serotype. Our results provide the basic knowledge for bioengineering the temperate phages that infect *S. enterica* serotypes, holding the potential to develop precise strategies for the prevention, surveillance, and management of this significant public health concern. Additionally, gaining a comprehensive understanding of the various serotypes, their prevalence, and the clinical manifestations associated with *S. enterica* infection by temperate phages can aid in the development of targeted approaches to address the diverse public health concerns caused by different *S. enterica* serotypes.

Moreover, it is important to acknowledge the limitations of our study. Firstly, there may be potential biases in the data due to the majority of phages being acquired from the U.S. and the U.K., which could limit the generalizability of our findings to a global scale. Furthermore, our study focused on basic sequence characteristics, and further investigations are needed to elucidate the functional implications of these correlations and the underlying mechanisms involved. Future studies should incorporate a wider range of phage samples from diverse geographical locations and consider additional factors such as gene expression patterns and host interactions to gain a more comprehensive understanding of phage integration.

In conclusion, our study provides insight into *S. enterica* temperate phages and their integrases and sheds light on their genetic characteristics and integration specificity. This work will facilitate future research into the interactions between phages and *S. enterica* and efforts to engineer phages to generate functional materials to better combat *S. enterica* infections.

## Data availability statement

The data and codes for data analysis in our study are available at the following GitHub repository: https://github.com/lindaandme/sqsun1001-phage.

## Author contributions

XZ conceptualized and designed the project, providing guidance throughout all aspects of this study. SS carried out the project under XZ supervision. All authors contributed to the article and approved the submitted version.

## Funding

This work was supported by the National Natural Science Foundation of China 31900489.

## Conflict of interest

The authors declare that they have no competing interests that could have influenced the design, conduct, or interpretation of this study.

## Publisher’s note

All claims expressed in this article are solely those of the authors and do not necessarily represent those of their affiliated organizations, or those of the publisher, the editors and the reviewers. Any product that may be evaluated in this article, or claim that may be made by its manufacturer, is not guaranteed or endorsed by the publisher.

## Footnotes

^1^http://ncbi.nlm.nih.gov

^2^https://www.chiplot.online/tvbot.html
