# Corrigendum: Genetic characteristics and integration specificity of *Salmonella enterica* temperate phages

**DOI:** 10.3389/fmicb.2023.1273462

**Published:** 2023-09-18

**Authors:** Siqi Sun, Xianglilan Zhang

**Affiliations:** ^1^State Key Laboratory of Pathogen and Biosecurity, Beijing Institute of Microbiology and Epidemiology, Beijing, China; ^2^Department of Life Sciences and Technology, Beijing University of Chemical Technology, Chaoyang, Beijing, China

**Keywords:** temperate phage, *Salmonella enterica*, integrase, attachment site, correlation analysis, phylogenetic analysis, taxonomic classification

In the published article, (Zhang et al., 2022) was not cited in Section **2.1** and the legend of **Figure 3**.

The citation has now been inserted in **2.1. Identification of integrase sequence**, paragraph 1 and should read:

“In the previous study, we first used FastQC (Babraham Bioinformatics, 2010) and Trimmomatic (Bolger et al., 2014) for quality control and NGS reads filtering, followed by the steps of prophage detection and temperate phage detection (Zhang et al., 2022).”

The citation has now been inserted in the legend of **Figure 3** and should read:

“These temperate phage genome sequences are from the database we generated in a previous study (Zhang et al., 2022).”

In the published article, (Bin Jang et al., 2019) was not cited in Section **3.4**.

The citation has now been inserted in **3.4. Taxonomic classification of** ***Salmonella enterica*** **temperate phages**, paragraph 1 and should read:

“In particular, we used vContact2 (Bin Jang et al., 2019) to cluster the temperate phage genome sequences with the reference virus genomes…”

In the published article, there was an error in the legend for **Figure 5** as published. The square brackets should be absolute values. The corrected legend appears below.

**Figure 5**. Distribution of distances between integrase sequences and attachment site core region sequences. Distance (integrase, core region) = |Position (integrase) – Position (core region)|/Length (temperate phage).

In the published article, there was an error in the keywords. The keyword “attachment” should be revised to “attachment site.”

In Section **2.4. Phylogenetic analysis of temperate phage and integrase entries**, paragraph 1, there was an error. The phrase “a neighbor-joining (NJ) tree” has been corrected to “the neighbor-joining trees”.

In Section **3.5**. ***Salmonella enterica*** **serotypes infected by the** ***Salmonella enterica*** **temperate phage genome sequences**, paragraphs 2 & 3, there were errors. The locations of most phages/integrases/core regions within the ranges were incorrectly stated. These paragraphs previously stated:

“Regarding the sequence length and GC content, we observed a relatively narrow range for *S. enteritidis* temperate phages, integrases, and core regions (**Figure 9A; Supplementary Table 9**). Most temperate phages had lengths between 39,267 and 39,280 bp, integrases ranged from 1,275 to 1,305 bp, and core regions spanned 81 to 87 bp. Similarly, their GC content exhibited a narrow range, with temperate phages ranging from 41.1% to 41.3% GC content, integrases from 48.9% to 49.0%, and core regions from 49.3% to 50.0% (**Figure 9B; Supplementary Table 9**).

For *S. typhimurium* temperate phages, their sequence lengths primarily fell within the 42,100 to 42,594 range, integrases ranged from 1,170 to 1,227, and core regions from 18 to 58 (**Figure 10A; Supplementary Table 10**). The GC contents of temperate phage genomes, integrases, and core regions were mainly between 50.0% to 50.2%, 46.7% to 48.5%, and 39.1% to 55.6%, respectively (**Figure 10B; Supplementary Table 10**).”

The corrected paragraphs appear below:

“Regarding the sequence length and GC content, we observed a relatively narrow range for *S. enteritidis* temperate phages, integrases, and core regions (**Figure 9; Supplementary Table 9**). Most temperate phages had lengths between 39,267 bp and 39,279 bp, integrases had a length of 1,275 bp, and core regions had a length of 81 bp (**Figure 9A; Supplementary Table 9**). Similarly, their GC content exhibited a narrow range, with most temperate phages ranging from 41.32% to 41.33% GC content, integrases at 49.02%, and core regions at 49.38% (**Figure 9B; Supplementary Table 9**).

For *S. typhimurium* temperate phages, their sequence lengths primarily fell within the 38,866 bp to 42,575 bp, integrases ranged from 1,173 bp to 1,227 bp, and core regions from 18 bp to 58 bp (**Figure 10A; Supplementary Table 10**). The GC contents of temperate phage genomes, integrases, and core regions were mainly between 50.05% to 50.23%, 46.70% to 51.18%, and 39.13% to 55.56%, respectively (**Figure 10B; Supplementary Table 10**).”

In Section **4. Discussion**, paragraph 6, there was an error. The percentages of different integrase types were incorrect. The sentence previously stated:

“Our analysis identified 3,857 unique *S. enterica* temperate phage sequences with integrases from three categories: intA (11.9%, 1,047/3,857), intS (31.9%, 2,801/8,777), and phiRv2 (0.1%, 9/3,857).”

The corrected sentence appears below:

“Our analysis identified 3,857 unique *S. enterica* temperate phage sequences with integrases from three categories: intA (27.15%, 1,047/3,857), intS (72.62%, 2,801/3,857), and phiRv2 (0.23%, 9/3,857).”

In the Section **3.4. Taxonomic classification of** ***Salmonella enterica*** **temperate phages**, paragraph 3, the last sentence has been deleted, because it is identical to the title of the following section.

The corrected sentence appears below:

“The taxonomic results show that from the aspect of integrase types, some temperate phages with intA and intS integrases were taxonomically clustered, respectively (**Figure 7**, the two clusters on the right; **Supplementary Tables 6, 7**); while some phages with different integrase types were taxonomically mixed together (**Figure 7**, left and middle; **Supplementary Tables 6, 7**). We conducted an analysis of the nucleic acid sequences of the core regions of *attP* and *attB* sites (**Figure 8; Supplementary Table 1**). The findings reveal that temperate phages with intA integrase exhibit consistent core regions at positions 18 to 21 (TGAG), while those with intS integrase show conservation at starting positions with AAT, ending positions with TA, and middle positions from 19 to 24 (TGCAGG). Furthermore, phages with phiRv2 integrase display consistency at all positions. These results highlight a correlation between the core region sequences and the integrase types, as evidenced by distinct conserved positions for different integrase types.”

The authors apologize for these errors and state that these do not change the scientific conclusions of the article in any way. The original article has been updated.

