# Supplementary material for: Genetic characteristics and integration specificity of Salmonella enterica temperate phages
Source: Front Microbiol. 2023 Aug 1;14:1199843. doi: 10.3389/fmicb.2023.1199843 (PMC10428622; doi:10.3389/fmicb.2023.1199843)
Supplement: Supplementary file 1 [file Data_Sheet_1.zip › Supplementary tables captions.DOCX]

Supplementary Material

Article Title

Siqisun^1^

Correspondence:

Corresponding Author: ^1^ Xianglilan Zhang, ^2^Ao liu

zhangxianglilan@gmail.com

# Supplementary Figures and Tables

## Supplementary Tables

**Supplementary Table 1.** Basic sequence characteristics of S. enterica temperate phages. The first column shows the SRA numbers of the hosts. Pos (integrase) indicates the position of the integrase sequence on the phage sequence, in the format of start position – end position. Dist (integrase-core region) indicates the relative distance between the integrase sequence and the core region sequence. Dist (integrase, core region) = (position of the integrase – position of the core region)/length of the phage.

**Supplementary Table 2.** Sample information of S. enterica temperate phages. "Sample" displays the SRA number of the host. "Collection_date" indicates the year it was sampled. "Host_original" indicates the host name originally uploaded to the public database. "Host_new classification" classifies the host into nine main categories: Animal, Environment, Feces, Food, Homo sapiens, Aquatic products, Plant, Water, and others. "Graphic location" indicates the country where the host was collected.

**Supplementary Table 3.** Information of all 3,857 unique S. enterica temperate phage entries. "Cluster" represents the cluster ID of each entry. "Integrase type" shows the type of the integrase included in this phage entry. "Length" and "GC content" illustrate the sequence length and GC content of the phage entry, respectively.

**Supplementary Table 4.** Information Information of all 491 unique integrase entries. "Cluster" represents the cluster ID of each integrase entry. "Integrase type" shows the type of this integrase entry. "Length" and "GC content" illustrate the sequence length and GC content of the integrase entry, respectively.

**Supplementary Table 5.** The correlation between S. enterica temperate phage entries and their integrase entries. The first column indicates the cluster ID number of each integrase entry. The second column indicates the number of temperate phage entries having this integrase entry. The last column indicates the cluster ID number of the temperate phage entries which have the integrase entry.

**Supplementary Table 6.** Structural characteristics for constructing the *S. enterica* temperate phage gene-sharing network, which was generated by vContact2.

**Supplementary Table 7.** Classification results of *S. enterica* temperate phages. The first column displays the phages in the database with the highest sequence similarity to our S. enterica temperate phages. The second column indicates the family to which these phages in the database belong.

**Supplementary Table 8.** Serotype information of temperate phages. The first column represents the serotypes, and the second column represents the number of temperate phages classified under each serotype.

**Supplementary Table 9.** Basic sequence characteristics of temperate phages classified as serotypes of S. enteritidis.

**Supplementary Table 10.** Basic sequence characteristics of temperate phages classified as serotypes of Typhimurium.
